# Influence of Degassing Treatment on the Ink Properties and Performance of Proton Exchange Membrane Fuel Cells

**DOI:** 10.3390/membranes12050541

**Published:** 2022-05-22

**Authors:** Pengcheng Liu, Daijun Yang, Bing Li, Cunman Zhang, Pingwen Ming

**Affiliations:** Clean Energy Automotive Engineering Center, School of Automotive Studies, Tongji University, Shanghai 201804, China; 2011683@tongji.edu.cn (P.L.); libing210@tongji.edu.cn (B.L.); zhangcunman@tongji.edu.cn (C.Z.); pwming@tongji.edu.cn (P.M.)

**Keywords:** catalyst ink, PEMFC, rheology, catalyst layer, impurity

## Abstract

Degradation occurs in catalyst inks because of the catalytic oxidation of the solvent. Identification of the generation process of impurities and their effects on the properties of HSC ink and LSC ink is crucial in mitigating them. In this study, gas chromatography-mass spectrometry (GC-MS) and cyclic voltammetry (CV) showed that oxidation of NPA and EA was the primary cause of impurities such as acetic acid, aldehyde, propionic acid, propanal, 1,1-dipropoxypropane, and propyl propionate. After the degassing treatment, the degradation of the HSC ink was suppressed, and the concentrations of acetic acid, propionic acid, and propyl propionate plummeted from 0.0898 wt.%, 0.00224 wt.%, and 0.00046 wt.% to 0.0025 wt.%, 0.0126 wt.%, and 0.0003 wt.%, respectively. The smaller particle size and higher zeta potential in the degassed HSC ink indicated the higher utilization of Pt, thus leading to optimized mass transfer in the catalyst layer (CL) during working conditions. The electrochemical performance test result shows that the MEA fabricated from the degassed HSC ink had a peak power density of 0.84 W cm^−2^, which was 0.21 W cm^−2^ higher than that fabricated from the normal HSC ink. However, the introduction of propionic acid in the LSC ink caused the Marangoni flux to inhibit the coffee ring effect and promote the uniform deposition of the catalyst. The RDE tests indicated that the electrode deposited from the LSC ink with propionic acid possessed a mass activity of 84.4 mA∙mg_Pt_^−1^, which was higher than the 60.5 mA∙mg_Pt_^−1^ of the electrode deposited from the normal LSC ink.

## 1. Introduction

Proton exchange membrane fuel cells (PEMFCs) have received significant research attention in recent decades, due to their high efficiencies, low operation temperature, and zero emissions [1,2,3,4]. Membrane electrode assemblies (MEAs), which comprise a proton exchange membrane (PEM), cathode and anode catalyst layers (CLs), microporous layers (MPLs), and gas diffusion layers (GDLs), are considered to be the heart of PEMFCs [5]. The complete working principle of an MEA consists of the following process: the oxidation reaction of H_2_ at the anode catalyst layer (ACL) provides electrons to an external circuit and releases protons to the internal electrolyte, while the reduction reaction of O_2_ at the cathode catalyst layer (CCL) receives electrons (from the external load) and protons (from the internal electrolyte). Both the CCL and ACL of an MEA are critical components of the system, because they represent energy conversion sites, where charge and mass transfer and the electrochemical reaction occur coinstantaneously [6]. The cost, performance, and durability of PEMFCs are closely dependent on the structure and morphology of CLs, which face several challenges, such as the coupling effects of corrosion in a strong acid environment, humidity stress, thermal shock stress, and mechanical stress during the service period [7]. Therefore, the optimization of the CL microstructure is a considerably critical issue to ensure a high performance of PEMFCs. 

Understanding and optimizing the preparation process of MEAs are imperative to obtaining CLs with the perfect microstructure for the most effective PEMFCs [8,9]. Generally, the process of preparing CLs involves the following procedures: (i) dispersion of the catalyst (such as Pt-loaded carbon) and the proton-conductive ionomer, which also works as a binder in a dispersion medium (such as water/NPA/isopropanol), in the CLs; (ii) coating of the catalyst ink on the PEM or GDLs; and (iii) drying to evaporate the dispersion medium [6,10]. The effects of the catalyst ink quality and process control exist throughout the above processes, which determine the CL microstructure and therefore the characteristics of the fuel cells [11]. Previous works have focused on the construction and optimization of the CL microstructure based on the coating process and ink formulation [5,12]. Coating and drying parameters influence the distribution of materials and pores in CLs and also have significant impacts on performance. Commonly, the ink formulation, including the alcohol content and type, ionomer content, and Pt dispersion, also affects the ink initial properties such as rheology, stability, and coatability, thereby exerting an influence on the fabrication of CLs [13,14,15,16]. However, the degradation of the catalyst ink quality after preparation also affects the catalyst ink viscosity, the size of agglomerates, which are a mixture of the catalyst and ionomer, the quality of the coated catalyst layer, and thus the performance of the fuel cells. Therefore, it is crucially necessary to understand the degradation process of catalyst inks and its impacts on the storage and rheologic properties, as well as well-constructed CLs. 

Based on extensive studies on CLs, many researches have demonstrated that the microstructure of CLs was closely realated to the catalyst properties, which was dependent on the size of agglomerates [11]. Catalyst particles are generally found to be agglomerated, forming primary agglomerates with a particle size of 200–300 nm under the effect of van der Walls attractive force. Further agglomeration of primary aggregates happens to generate secondary agglomerates on the microscale. Additionally, the addition of an ionomer can reduce the size of agglomerates due to the electrostatic repulsion and steric hindrance interactions. The larger agglomerates become, the greater negative effect they will have on the construction of CLs, leading to a reduced output performance. Therefore, the state of the catalyst ink should be controlled at a uniform and stable condition to fabricate high-quality MEAs.

The change in composition of the catalyst ink will lead to a change in its properties, especially the generation of impurities. To date, only a handful of studies have described the generation of impurities in catalyst inks and their effects on the processability of catalyst inks [14,17,18]. For instance, some previous works have demonstrated the effects of various impurities, such as acids and aldehydes, on the agglomerate behavior of inks and ultimately on the final structure of CLs [14,17,18,19,20]. Uemura [14,18,19] used X-ray computed tomography to detect the presence of air bubbles and the third phase in a catalyst ink and proved the catalyst caused alcohol to decompose [19]. Kameya combined nuclear magnetic resonance (NMR) with magnetic resonance imaging (MRI) to analyze the internal state of an ink during the preparation process and detected the presence of air bubbles in the ink during the main mixing process [21]. In addition, ^19^F NMR spectra revealed dramatic changes in the dispersion states of Nafion during the mixing period. Other previous studies targeting NPA oxidation on platinum electrodes in acid solutions have demonstrated that NPA is converted to propionic acid, whereas isopropanol is highly selectively converted to acetone, due to the difficulty in breaking the C−C bond [22,23]. Catalyzed oxidation of the dispersion medium and the deuterogenic reaction affect the state of the catalyst ink. These generated impurities induce the generation of larger agglomerates in the catalyst ink, and thus cracking of the CLs because of the capillary stress. Kumano [13] identified the structural parameters that control the dispersion state and stability of Pt/C agglomerates. In inks containing 48–75% of water, the amount of adsorbed ionomers decreased with decreasing water content, resulting in increases in the viscosity, storage modulus, and electrical conductivity. The adsorption rate of the ionomer into the Pt/C decreased, and the average size of agglomerates, viscosity, and storage modulus increased with the increase in the hydrophobicity of the solvent. The impurity produced in inks undoubtedly changes the hydrophobicity of the solvent and thus affects the properties of the ink. Hence, it is important to obtain an understanding of the generation process of impurities and their effects on processability. 

HSC ink is often used in the coating procedure across industrial applications, whereas LSC ink is applied in the spraying procedure and rotating disk electrode (RDE) tests in laboratories [13,24]. High-quality RDE measurements need a thin, uniform film over the entire surface area of the glassy carbon to accurately evaluate the electroactivity of the catalyst [24]. The quality of the working electrode is delicately determined by the drying conditions, alcohol content and type, Pt dispersion, and surface state of the glassy carbon. Therefore, the effects of impurities on the properties of LSC ink require a detailed investigation, owing to their effect on the electrode structure, and the lack of clarity regarding their underlying mechanism of action. At present, there is limited cognition of the formation mechanism of impurities and their effects on the catalyst ink rheology and drying behavior. To fully explore the generation process of impurities, we first investigated the oxidation of the solvent and the effects of the temperature and atmosphere on catalyst ink degradation. In order to understand how impurities affect the rheology and drying process of catalyst inks, we present a comparative study of HSC ink before and after degassing treatment. The range of comparison includes rheological behavior and the property (ink)–structure (catalyst layer)–performance (MEA) relationship.The influence of impurities on the properties of LSC ink cannot be ignored because the LSC ink is widely used in the RDE experiments to evaluate the characteristics of catalysts. The effects of the introduction of propionic acid in the LSC ink on the microscale structure of the RDE were investigated using optimal microscopy, cyclic voltammetry (CV), and line sweep voltammetry (LSV). Based on the understanding of the impurity evolution process and the relationship between the impurity properties and ink quality for the two ink types, we provide insight into optimizing the preparation of catalyst inks to obtain excellent processability and coatability. This also helps in the construction of the desired catalyst layers.

## 2. Experimental Procedures

### 2.1. Preparation of Catalyst Ink 

**Figure 1:**
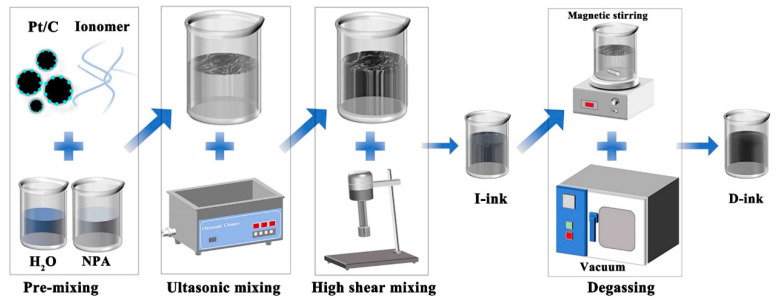
Schematic representation of the process of HSC ink preparation.

This section describes the procedures and instrumentation for the preparation of the HSC ink. Briefly, 5.7 g of Pt/C powder (Johnson Matthey, Alfa Aesar, Shanghai, China, Vulcan XC-72 with 60 wt% Pt and 20.5 g of Nafion^®^ solution (Dupont^™^, New Castle, DE, USA, Nafion^®^ PFSA polymer dispersions D-520) were mixed with 27 g of the NPA and ultrapure water (the ratio of NPA to ultrapure water was 1) dispersion medium. The process of fabricating the HSC catalyst ink followed the steps shown in Figure 1. Firstly, 5.7 g of catalyst powder was added to 13.5 g of water and stirred with a glass rod, and then 13.5 g of NPA and 20.5 g of Nafion solution were added successively (premixed process), followed by ultrasonic dispersion (35 kHz, 5 min, 15 °C). Secondly, the catalyst ink was homogenized by high-speed shear (1600 rpm, 30 min) and finally degassed using a magnetic stirrer at 30 rpm for 30 min at −0.1 MPa. The ink and raw material for the above process were contained in glass containers. The catalyst ink that was not degassed was measured after being kept for 24 h and marked as “I-ink”, whereas the degassed ink was denoted “D-ink” and left to stand for at least 24 h before subsequent measurement. After preparation of the two catalyst inks, the container was filled with nitrogen as the protective gas.

The LSC ink for oxygen reduction reaction (ORR) catalyzed activity was evaluated using a rotating disk electrode (RDE). Briefly, 2 mg of catalyst powder was added to a 1 mL mixture of 5 wt% Nafion^®^ solution and NPA (volume ratio of 1:30), and the mixture was fully mixed by ultrasonication for 40 min (35 kHz, 15 °C). This LSC ink was denoted “N-ink”. To characterize the effect of impurities on the LSC ink, we added 10 μL of propionic acid (playing the role of impurities) to the N-ink to produce a comparison ink known as “P-ink”. A summary of the compositions of the HSC ink and LSC ink is shown in Table 1.

### 2.2. Electrochemical Evaluation 

The HSC catalyst ink was directly coated on the proton exchange membrane (Gore, Newark, DE, USA, thickness of 18 μm) using a slot-die coating system. The slot die moved at a horizontal velocity of 10 mm/s above the proton exchange membrane with a coating gap of 100 μm. The baseplate temperature was maintained at 60 °C to remove the solvent from the wet film. The MEA was assembled by sandwiching a catalyst coating membrane between two pieces of gas diffusion layer (Freundenberg, Shanghai, China, H24CX483). The Pt loadings were controlled to be 0.4 mg∙cm^−2^ and 0.2 mg∙cm^−2^ in the cathode and anode, respectively. To evaluate the fuel cell performance, the polarization curve was measured with a 25 cm^2^ three-serpentine cell fixture and tested with a fuel cell test system (Dalian New Sunrise Testing Technology Co., Ltd., Dalian, China, NSR-FTCS100B-1802-3). The temperature of cell was controlled at 75 °C. The stoichiometry of H_2_/air was 1.5/2.5. The inlet gauge pressures of anode and cathode were maintained at 100 and 80 kPa. The relative humidity at the anode and cathode sides were both 55%. The electrochemical impedance spectra of single cells were recorded at 1.6 A·cm^−2^ using a scanning frequency from 10^3^ to 0.1 Hz. A detailed electrochemical analysis of the oxidation behavior of NPA and EA under an acid environment was performed using a three-electrode cell system (PINE, AFCPRBE). This system comprises a thin film of catalyst ink deposited on a glassy carbon substrate as the working electrode (WE), a reversible hydrogen electrode (RHE) as the reference electrode, and a platinum sheet as the counter electrode. The electrolyte solution was de-aerated before each measurement with N_2_ and O_2_ for 30 min, and all electrochemical measurements were performed using an electrochemical workstation (CHI. Instrument company, Shanghai, China, 760E). The working electrode was prepared by transferring 10 μL of ink into the RDE (S = 0.196 cm^2^), followed by natural drying. CV and LSV were carried out in 0.1 M HClO_4_ at 25 °C. CV data were recorded at a potential range from 0.05 to 1.10 V, at a scanning rate of 0.05 V·s^−1^, in a N_2_-saturated electrolyte solution. ORR polarization curves were obtained in an O_2_-saturated electrolyte solution at a scanning rate of 0.005 V·s^−1^ and an RDE rotation rate of 1600 rpm. The electrochemical surface area (ECSA) of the WE was calculated based on the CV curves, using the following equation [25]:

(1) $$\mathrm{ECSA}=\frac{Q_{H}}{210\times m_{\mathrm{Pt}}}$$

where Q_H_ (mC) is the charge of hydrogen species’ electro-adsorption peak; the value of 210 μC·cm^−2^ corresponds to monolayer adsorption of hydrogen atoms on a polycrystalline Pt; and m_Pt_ represents the mass load of Pt on the working electrode. ORR’s catalytic activity, which is the kinetic current density at 0.9 V (vs. the RHE) from the LSV curve, was calculated based on the Koutecky–Levich (K–L) equation as follows [26]:

(2) $$\frac{1}{i}=\frac{1}{i_{k}}+\frac{1}{i_{d}}$$

where *i* is the current density value measured at E = 0.9 V; *i_d_* is the diffusion-limited current density at E = 0.4 V (vs. the RHE); and *i_k_* represents the kinetic current. The specific mass activity (MA) of the catalyst is the kinetic current per unit mass loading of Pt [24,27].

### 2.3. GC-MS Instrumentation

Impurities in the catalyst ink were analyzed by GC-MS (Agilent, Shanghai, China, 7890B-5977B), which can detect various volatile components in a solution. Patterns of the mass spectra were analyzed using NIST-2008.

### 2.4. Rheological Measurements

The rheological property of the inks was measured using a stress-controlled rheometer (Anton Paar, Shanghai, China, MCR302), with a coaxial cylinder mold. Prior to measurement, the ink was kept quiescent at 25 °C for 5 min to remove any previous disequilibrium status and ensure that the constituent material established new equilibrium-status structures. A pre-shear treatment was first used to eliminate the shear history and ensure the repeatability of the test data. During this operation, the shear rate was controlled at 0.01 s^−1^ for 100 s. Thereafter, steady status flow measurements were carried out by step-wisely increasing the shear rate from 0.01 to 1000 s^−1^, to test the viscosity function of the formulated inks. Three interval thixotropy tests were used to determine the structural regeneration of the HSC ink, and a typical step test with three intervals depicted as a time-dependent viscosity function was as follows: (1) the shear rate was kept at 0.1 s^−1^ for 60 s, at the beginning, to simulate the ink at rest; (2) the shear rate was maintained at 100 s^−1^ for 10 s to simulate the structural breakdown of the ink; (3) the rate was kept at 0.1 s^−1^ for 60 s to simulate the structural recovery of the ink at rest. Furthermore, the strain dependency of the storage modulus (G′) and loss modulus (G″) was applied to change the strain from 0.01 to 100% at 1 Hz after tests of the steady flow viscosity. All rheology experiments were performed at 25 ± 0.1 °C.

### 2.5. Measurement of Ink Cluster Size and Zeta Potential

The cluster size and zeta potential measurements of HSC were performed with dynamic light scattering (DLS) (Colloid Metrix, Shanghai, China, Nano-fiex) and a particle potential titrator (Colloid Metrix, Shanghai, China, Stabino), respectively. For testing purposes, 0.1 mL of the inks was diluted using 100 mL of a solution with the original solvent composition. The diluted inks were dispersed in an ultrasonic bath for 2 min prior to the DLS and zeta potential measurements.

### 2.6. Determination of Contact Angle and Deposition of LSC Ink

The morphology of the LSC ink (5 μL) drying on the RDE was measured using a digital microsystem (KEYENCE, Osaka, Japan, VH-S30B). The contact angle between the catalyst ink and the glass was determined, using the side view of the microscope, and the deposition process of the catalyst ink droplets was observed from the top view.

## 3. Results and Discussion

**Figure 2:**
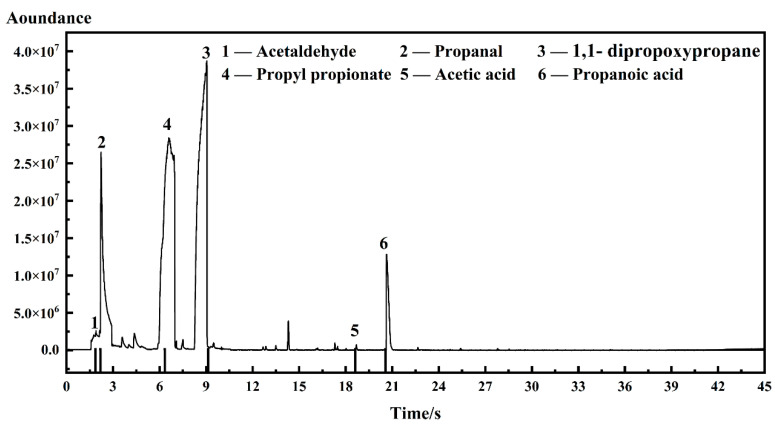
Total ion current (TIC) spectrum of I-ink.

**Figure 3:**
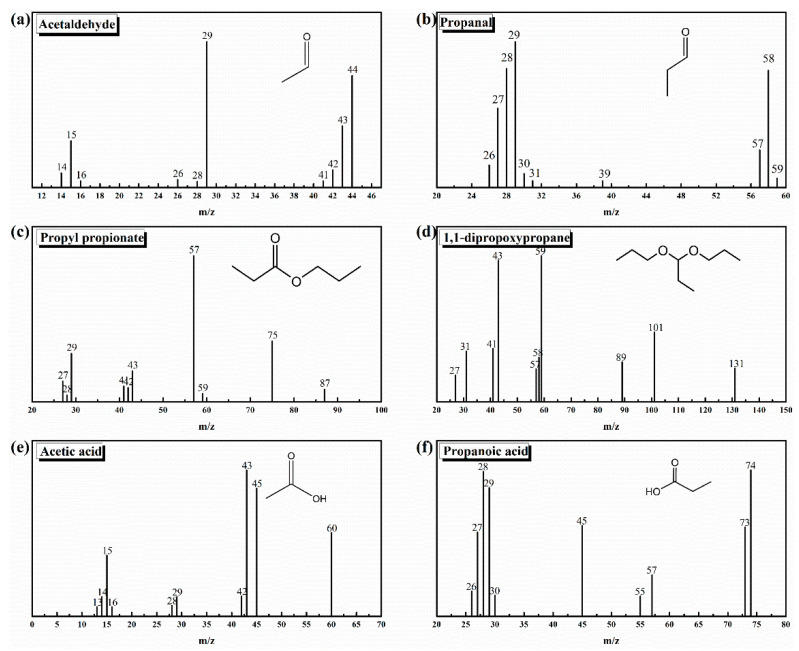
Mass spectra of impurities derived from electron impact ionization of I-ink: (**a**) acetaldehyde; (**b**) propanal; (**c**) propyl propionate; (**d**) 1,1-dipropoxypropane; (**e**) acetic acid; (**f**) propanoic acid.

The total ion current (TIC) spectrum of I-ink, based on GC-MS detection, and the spectra of D-ink are shown in Figure 2 and Figure S1; the signals of impurities classified by their corresponding mass spectra are illustrated in Figure 3; and the results of the quantitative analysis are listed in Table 2. 

The impurities in the ink were indexed as acetaldehyde, propanal, 1,1-dipropoxypropane, propyl propionate, acetic acid, and propanoic acid. The corresponding regions of the TIC spectrum were detected after 1.865, 2.194, 9.136, 6.338, 18.620, and 20.562 s. The presence of acetaldehyde was confirmed by the fragment ions (*m*/*z*) at 29, 43, 44, and 15 (Figure 3), whereas the propanal segments were ionized into *m*/*z* of 26, 27, 28, 29, 57, 58, and 59. Other impurities were identified through additional analysis of the mass spectra, such as propyl propionate, 1,1-dipropoxypropane, acetic acid, and propanoic acid. Interestingly, these impurities were found to simultaneously occur in I-ink and D-ink, albeit with significant differences in concentrations. Therefore, the degassing treatment efficiently suppressed the oxidation process of the solvent. Previous studies have demonstrated that Pt metal can catalyze solvents to produce complex oxidizing compounds, including acetaldehyde, propanal, acetic acid, and propanoic acid [22,28,29]. These catalytic products are then condensed to form esters. We observed significant differences in impurity concentrations between I-ink and D-ink. It is worth noting that the oxidation products exhibited more hydrophobic characteristics than the original solvent composition. Nafion^®^, a binder and stabilizer in catalyst inks, is essential for optimization of the properties of catalyst inks [30]. In fact, its hydrophobic backbone is attracted to the hydrophobic surface of the carbon support, whereas its hydrophilic sidechains are ionized to generate numerous ionic charges on the surfaces of the carbon support [31]. However, the presence of hydrophobic impurities improved the compatibility between the ionomer and solvent, thereby increasing the amount of free ionomer on the solvent. Consequently, this affected the interaction between the internal components and rheology of the catalyst ink [16,32]. Next, we investigated the effects of the atmosphere and temperature on the impurity generation process.

**Figure 4:**
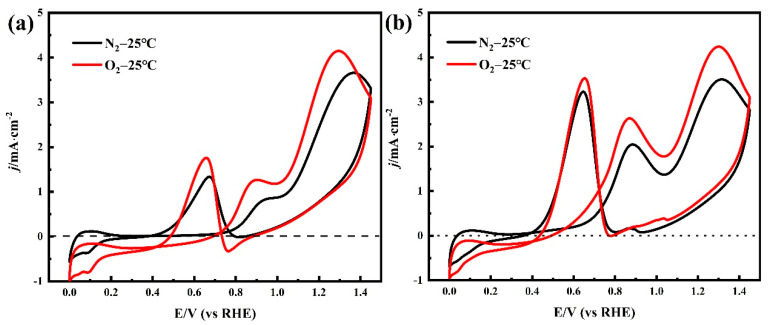
(**a**) Cyclic voltammograms recorded with a Pt/C electrode in 0.05 M H_2_SO_4_ + 0.1 M NPA solution and (**b**) 0.1 M EA solution, with a scan rate of 20 mV·s^−1^ and a temperature of 25 °C.

**Figure 5:**
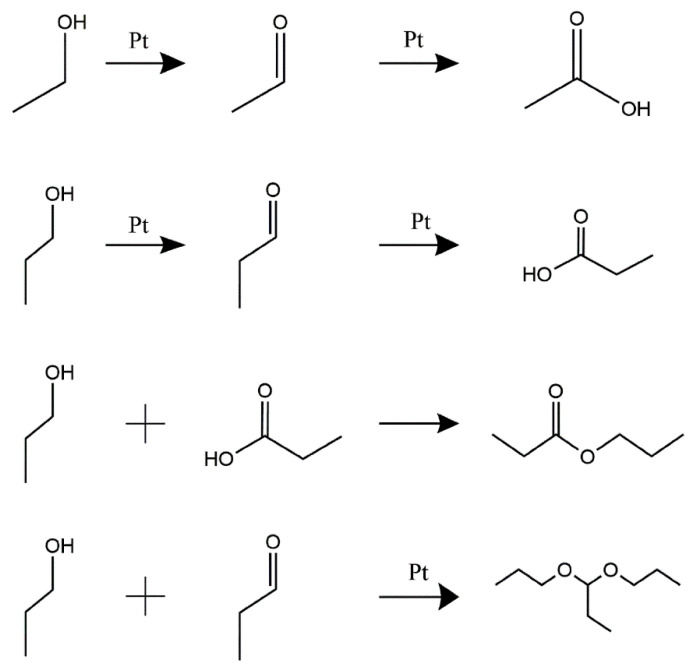
A schematic illustration of the proposed NPA and EA conversion process.

The CV of Pt/C electrodes in 0.05 M H_2_SO_4_ + 0.1 M NPA solution at 0.002 V·s^−1^ shows the electro-oxidation process of NPA (Figure 4a). The first oxidation peak was detected at 0.90V vs. the RHE in the O_2_-purged solution, in the positive scanning process. In contrast, this peak was found at 0.95 V vs. the RHE in the N_2_-purged solution, due to the overpotential required to overcome the concentration polarization caused by the lack of O_2_. Both oxidation peaks corresponded to the poisonous intermediate formation in the NPA oxidation reaction [28]. However, an increase in the potential generated the second oxidation peak at 1.29 V in the O_2_-purged solution, whereas a similar peak was observed at 1.35 V in the N_2_-purged solution. This oxidation peak indicated the formation of reaction intermediates during NPA oxidation. Furthermore, several higher peaks’ current densities were recorded in the O_2_-purged solution relative to the N_2_-purged solution, because sufficient electro-oxidation of NPA produces a larger reaction current. A similar phenomenon was observed during the oxidation of alcohol (Figure 4b). The oxidation peaks’ current densities of NPA and EA are summarized in Table 3. The high impurity concentration of I-ink derived from quantitative analysis of GC-MS also supported this phenomenon (Table 2). As previously mentioned, the CVs of NPA and EA in the O_2_ atmosphere exhibited a significantly higher oxidation overpotential and smaller oxidation current density, suggesting that the anoxic environment can reduce the intensity of solvent oxidation reactions. The effects of the temperature on the electro-oxidation of NPA and EA are discussed in the Supporting Information, and the CVs of NPA and EA under various temperatures are shown in Figure S2. Summarily, high temperatures promoted the solvent’s oxidation behavior, suggesting the need to regulate the N_2_ atmosphere and control the temperature for an effective reduction in solvent electro-oxidation during the ink preparation process. A summary of the mechanism underlying the formation of impurities in the catalyst inks is shown in Figure 5. Briefly, EA and NPA can be oxidized to their respective aldehydes and acids, in the presence of platinum catalysis; meanwhile, propyl propionate is generated by esterification of propionic acid and propanol. Specifically, the aldolization reaction of NPA and propanal, via Pt catalysis, is the cause of 1,1-dipropoxypropane [14]. 

**Figure 6:**
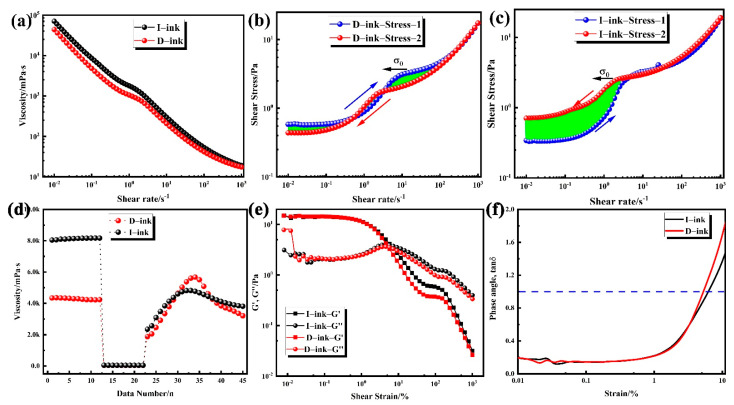
(**a**) Dynamic viscosity data of tested catalyst inks; (**b**,**c**) shear stress as a function of the shear rate for I-ink and D-ink; (**d**) three interval thixotropy test of catalyst inks, and amplitude oscillation test of the inks; (**e**,**f**) strain-dependent storage modulus (G′) and loss modulus (G″) at 1 Hz and phase angle.

Rheological characterization of the catalyst ink is an essential index for each step during MEA fabrication. The catalyst ink is taken as the working fluid in a slot die, and its viscosity is perceived as the most crucial rheological property during the coating procedure [33,34]. It directly influences the behavior of the ink formulation during mixing and production of the wet catalyst layer [35]. The relationship between viscosity and shear stress is shown in Figure 6a. Obviously, shear thinning behavior occurred in D-ink and I-ink, which means the viscosity was negatively correlated with the shear rate. Catalyst inks are multi-component, complex solid–liquid mixtures that consist of a catalyst, ionomer, and solvent medium. The dynamic viscosity at low shear rate stages is an index of the settling degree of the solid content, while that at high shear rate stages is an index of the coating processability [36]. Both inks showed a high viscosity at a low shear rate stage, which is preferred owing to the lack of significance in the settlement of the solid content. Notably, at a higher shear rate stage, the strong shear rate force tended to destroy the microstructure of the catalyst inks, which subsequently realigned the internal structure and significantly reduced the viscosity. This behavior means that D-ink and I-ink are non-Newtonian fluids, a property that is quite suitable for the actual production process. At the ink storage stage, the particles in the ink were subjected to external forces, including gravity and shear forces. The shear rate ranged from 10^−6^ to 10^−2^ s^−1^. The high viscosity indicates the excellent anti-sedimentation properties of these inks. During coating, the fluid with a high shear rate requires a low viscosity. After coating, the advection of ink occurs on the proton exchange membrane under surface tension and the action of gravity. The viscosity of I-ink was slightly higher than that of D-ink in most of the shear rate scope, but with the shear rate increasing, the gap between the viscosities of the inks gradually narrowed. This implies that the network structure of the catalyst and binder grew after degassing, and dispersion states in the ink were changed. The rising hydrophobicity of the solvent caused an increase in free ionomers, which subsequently increased the viscosity [37,38]. The difference in the network structure’s strength between these two inks was further evidenced by the hysteresis flow curves, as shown in Figure 6b,c. Notably, hysteresis phenomena, where different shear stress values appeared in the positive scan and negative scan, were observed in the inks. Moreover, any destabilization of this steady state would destroy the ionomer structure, owing to entanglements of ionomer chains and fluctuations in the arrangement of the catalyst particles brought about by changes in shear and relaxation processes [39,40]. The degassing treatment improved the adsorption of the ionomer into the catalyst, due to the removal of the microbubbles in the aggregates. 

Furthermore, the continuous increase in bridging within the catalyst, yield stress, level of shear thinning, and equilibrium G′ within the inks’ linear viscoelastic regime were all strengthened, and both types of inks exhibited a shear stress plateau at shear rates from 1 s^−1^ to 10 s^−1^, indicating the existence of yield stress in both inks (Figure 6b,c). We calculated the numerical value of the yield stress by averaging the initial five points in this stress plateau and found a higher value in I-ink (2.5 Pa) than in D-ink (1.7 Pa). The decrease in the yield stress of the inks contributed to their self-leveling, which suppressed the uneven thickness distribution in the catalyst layer [5,41].

According to the determined yield stress, the level of shear thinning of the inks can be quantified by implementing the Herschel–Bulkley model [42,43]:

(3) $${\sigma =\sigma }_{0\;}{+K\gamma }^{n}\;$$

where σ and $\sigma_{0}$ represent the measured shear stress and yield stress measured at a specific shear rate (Pa), respectively; K denotes the consistency index (Pa·s^n^); γ is the shear rate (s^−1^); and n is the dimensionless flow index. Only information obtained from a shear rate above 2 s^−1^ was considered in the modeling procedure, because this range of the shear rate matches the actual coating process. The consistency index indicates the degree of viscous contribution during the increase in the shear rate, whereas the function of shear stress and the shear rate of the catalyst inks were presented by the flow index [44]. As shown in Table 4, the results show that I-ink had a significantly higher consistency index than D-ink because I-ink exhibited a significantly higher level of shear thinning and was more viscous than D-ink. All of the inks had *n* < 1, a phenomenon that corresponds to the shear thinning behavior as illustrated in Figure 6a. Notably, a small dimensionless flow index resulted in stronger shear thinning behavior [42], and the gap in viscosity between D-ink and I-ink almost disappeared when the shear rates were increased to about 100 s^−1^. On the other hand, an increase in the low-shear viscosity (LSV) resulted in a coating layer with a sharper edge, implying less cut-off waste during subsequent processing [45].

Catalyst ink coating entails a high-shear-rate process, while self-leveling of the ink onto the PEM is a low-shear-rate process. The essential requirements for this structural regeneration process include: (1) applying a slow reconstruction rate for good leveling; and (2) ensuring the rate is not too slow to prevent sagging and to allow a sufficient wet layer thickness and flatness. To investigate this time-dependent behavior, we performed a rotational test with three intervals and present the result as a time-dependent viscosity function (Figure 6d). At the first stage, a very low shear rate (0.1 s^−1^) was used to simulate behavior at rest, and as the hydrophobicity of the dispersion solvent increased, the viscosity of D-ink became lower than that of I-ink. This difference in viscosity resulted from the increase in the free ionomer and generated aggregation, which was related to the change in the rate of adsorption of the ionomer into the catalyst [13,38,46]. An increase in the shear rate to 100 s^−1^ (stage 2) caused the strong shear to simulate the structural breakdown of the catalyst inks during the coating process [47]. Moreover, both ink types exhibited very low viscosity due to the shear thinning behavior. At the final stage, the low shear rate simulated structural regeneration for the ink self-leveling process, although the structural strength and viscosity of the inks gradually recovered with time. The thixotropic recovery rates of D-ink and I-ink were 75.8 and 46.6%, respectively. We hypothesized that the high ionomer adsorption on the catalyst’s surface strengthened the interaction between the ionomer and the catalyst, and this behavior was also observed in the lithium-ion battery field [47,48].

Next, we used oscillatory shear to investigate the inks’ microstructure, and amplitude sweep to characterize the inks’ linear viscoelastic regime (LVR). Subsequently, we applied the LVR to accurately measure the breakdown of the network structure and acquire the structural strength of the initial state [42]. The results reveal lower G″ values for both inks at the low-strain region compared to G′, indicating an elastic-dominant response property of the inks (Figure 6e) [42]. At a strain range of 5% to 7%, the G′ value fell below that of G″, indicating that only a slight increase in the shear strain could promote the inks’ shift from elastic-dominant to viscous-dominant [49]. The phase angle δ of the ink, which is calculated using Equation (4) below, is shown in Figure 6f.

(4) $${\delta =\tan}^{-1}\left(\frac{G^{\prime \prime }}{G^{\prime }}\right)$$

Briefly, the value of δ for D-ink reached 1 at a faster rate, indicating that the gel–sol transformation occurred more easily. For D-ink, the better self-leveling effect, during the drying procedure, resulted in the gradual development of pores in the wet catalyst layer. Therefore, a homogenized cavity structure is beneficial to the reduction in capillary stress during the drying process. In contrast, a wet film with low self-leveling after drying generates a hierarchic pore structure with larger fluctuations, thereby enhancing capillary stress and increasing the risk of CL cracks [46,50,51,52,53].

**Figure 7:**
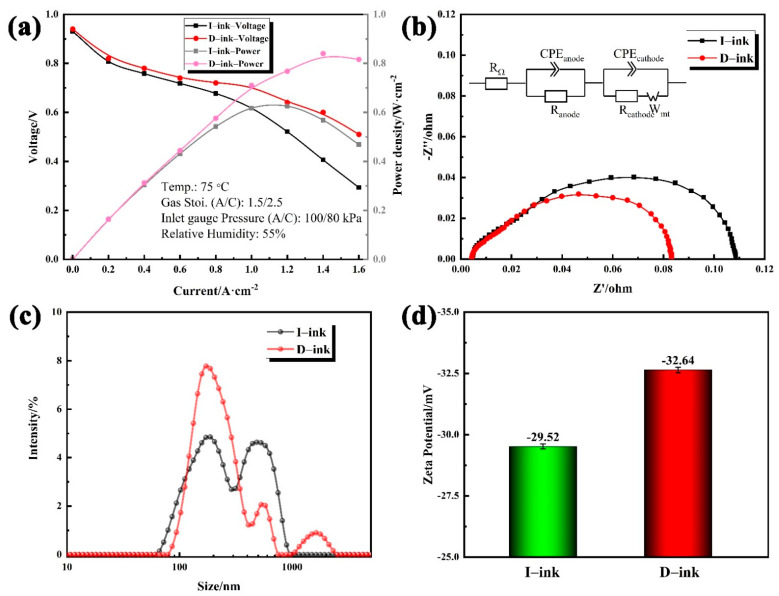
(**a**) H_2_/air polarization curves of membrane electrode assemblies fabricated with HSC inks, and (**b**) corresponding Nyquist plots obtained at 1.6 A∙cm^−2^ from 0.1 Hz to 1 kHz; (**c**) size distribution and (**d**) zeta potential of I-ink and D-ink.

Figure 7a shows the cell polarization curves of the MEAs fabricated from different HSC inks. According to Figure 7a, the MEA prepared from D-ink exhibited excellent improvements compared with that made from I-ink. At the electrochemical polarization control region, the voltage of the two MEAs showed no distinct differences, which can be attributed to the catalyst having the same catalytic intrinsic activity in both MEAs. With the increase in the current density, the gap in output voltages of the different MEAs expanded. Especially under high current densities, mass transfer loss led to a significant performance reduction for the MEA fabricated with I-ink. The electrochemical impedance spectra were recorded to analyze the H_2_/air performance and fitting using an equivalent circuit (Figure 7b) [54]. *R*_Ω_ denotes the ohmic resistance of the cell. *R*_anode_ and *R*_cathode_ are faradaic resistances, which represent the kinetics of the electrochemical reactions occurring on the anode and cathode, respectively. The finite Warburg circuit element (*W*_mt_) is used to reflect the mass transport loss on the cathode side. As observed, the MEA performance improved with the decrease in the impedance arc. The fitted values of *R*_Ω_ were 0.0043 Ω and 0.0045 Ω for D-ink and I-ink, respectively. The impedance spectra consist of semicircles in the high-, medium-, and low-frequency regions, and each of these semicircles corresponds to the resistances of anode activation, cathode activation, and mass transport. The increments in the semicircles in the medium- and low-frequency regions reflect the greater resistance of the activation kinetics and mass transport. The *R*_anode_ values for D-ink and I-ink were similar, with 0.0211 Ω and 0.0223 Ω, respectively, due to them having the same anode catalyst layer. The MEA fabricated with I-ink exhibited a higher *R*_cathode_ and *R*_mt_ than that fabricated with D-ink. The *R*_cathode_ values decreased from 0.0604 Ω to 0.0353 Ω, and the *R*_mt_ values varied from 0.0255 Ω to 0.0183 Ω, with the degassing treatment of the ink. Therefore, the cathodic mass transport process and ORR kinetics dominated the H_2_/air performance. Jian Xie [12] reported a similar phenomenon where the increase in the NPA ratio in the solvent could intensify the resistance of the ORR kinetics and mass transport limitations. 

To better understand how the impurities affect the MEA performance, the catalyst cluster size distributions and zeta potential in different inks, which determine the catalyst/ionomer interface and CL structure, need to be studied. The microscale mass transport in the CLs depends on the aggregate structure and ionomer distribution [55]. As evidently shown in Figure 7c, the intensity signals in I-ink showed a double-peak structure, indicating quite a few of the larger clusters. On the contrary, the intensity signals of D-ink were concentrated in small-size regions. The average diameter of I-ink clusters reached 244.7 nm, while the size of the D-ink clusters was 199.7 nm. The zeta potential results show the stability differences for D-ink (−32.64 mV) and I-ink (−29.52 mV). A hydrophobic impurity causes the ionomer to adsorb into the solvent and desorb from the catalyst. This reduces the adsorption capacity of the ionomer, leads to the inhibition of steric hindrance between the clusters, and increases the risk of cluster agglomeration. 

**Figure 8:**
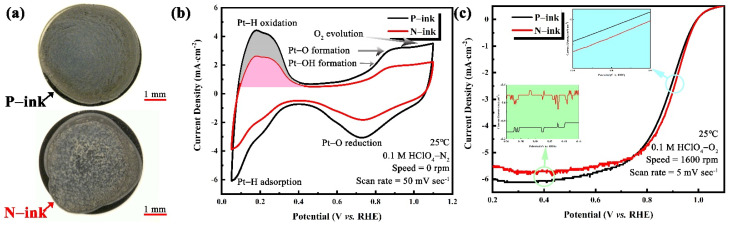
(**a**) Optical photograph and ORR performance of the electrodes in an RDE: (**b**) CV and (**c**) the corresponding ORR polarization curves.

Apart from electrochemical contamination, impurities also affect the drying behavior of LSC ink. High-quality ORR tests require a thin, uniform film over the entire surface area of the GC electrode [56,57,58]. However, the catalyst dispersed in a drying ink drop migrates towards the edge of the ink drop to form a “coffee ring” [59,60]. The effects of impurities on catalyst activity were tested in a half-cell using the as-prepared ink coating on a GC electrode. Deegan et al. [59,61,62] postulated that the “coffee ring” effect occurs because the evaporation rate at the edge of droplet is higher than that at the center, resulting in an outward capillary flow within the droplet. This, in turn, transfers the suspended particles to the edge of the droplet and deposits them into a ring at the edge. As shown in Figure 8a, the “coffee ring” phenomenon appeared in the N-ink electrode, leading to an ununiform distribution of the catalyst, but this phenomenon was alleviated in the P-ink electrode. Notably, the introduced propionic acid has a higher boiling point and lower surface tension than the original solvent. As evaporation proceeds, the water evaporation rate at the edge of the droplet exceeds that at the center, whereas the evaporation rate of the propionic acid at the edge of the droplet becomes slower. Therefore, the propionic acid gradually becomes enriched at the edge. The difference in surface tension between the edge and center of the droplet creates the Marangoni effect [63]. On this basis, the enhanced Marangoni flow moves the catalyst particles radially from the edge to the center of the droplet surface, thereby inhibiting the “coffee ring” effect. Therefore, an RDE with a uniform catalyst deposition layer shows a better ORR performance.

The CV curves of electrodes from N-ink and P-ink are shown in Figure 8b. The anodic H waves and the cathodic H waves in the CV represent the H from the electrochemical desorption and adsorption process, respectively [24,27,64]. The H_upd_ charge is estimated after the conventional correction for the pseudocapacity seen in the double-layer region by a straight line. As an electron is transferred during the oxidation of the adsorbed H_upd_, the charge of Hupd is therefore given by $Q_{H}=\frac{\int idE}{v}$ with the potential *E*, the sweep rate *v*, and the current *i*. The amounts of H_des_ in the curves of P-ink and N-ink, with double-layer charges subtracted, are 1.58 mC and 2.83 mC, respectively. In agreement with the report of Garsany et al. [24], the film quality affects the electrochemical surface area measurement, with an ECSA of 62.7 m^2^∙g_Pt_^−1^ for the electrode fabricated from N-ink, compared to 112.2 m^2^∙g_Pt_^−1^ for the electrode fabricated from P-ink (cf. Table 5—data from tables). Notably, the data shown in Figure 8c indicate that the electrode deposited by P-ink had a higher mass activity (84.4 mA∙mg_Pt_^−1^) than that deposited by N-ink. Furthermore, the normal LSC ink exhibited lower electrochemical properties of electrode deposition than the LSC ink with propionic acid, under similar conditions of the catalyst ink formation and drying process. The superior catalyst performance of the N-ink electrode was attributed to the morphology of the deposited catalyst, due to the effect of impurities on the ink [65]. The records of the drying process for N-ink and P-ink are presented in the Supporting Information to reveal the drying behavior of the ink droplets on the glass substrate.

## 4. Conclusions

In the traditional ink preparation procedure, the catalytic oxidation of alcohols and its effects on the quality of the catalyst ink are generally ignored. In this study, we explored the mechanism by which impurities are generated, and the effects of such impurities on HSC ink and LSC ink. The GC-MS results indicate that the impurities in the inks included propionic acid, acetic acid, propanal, acetaldehyde, propyl propionate, and 1,1-dipropoxypropane. Together with the electrochemical behavior of NPA and EA, the impurity evolution process is as follows: NPA and EA are catalytically oxidized to propionic acid and acetic acid. Then, they are under esterification and aldolization reactions. The strength of the catalytic reaction is strongly correlated with the oxygen atmosphere and temperature. This result demonstrates the importance of temperature control and degassing treatment during the ink preparation process.

The effects of impurities on the HSC ink and LSC ink were identified, and the production of impurities was suppressed by the degassing treatment, causing the concentrations of acetic acid, propionic acid, and propyl propionate to decrease from 0.0898 wt.%, 0.00224 wt.%, and 0.00046 wt.% to 0.0025 wt.%, 0.0126 wt.%, and 0.0003 wt.%, respectively. For the HSC ink, the viscosity and yield stress of D-ink were lower than those of I-ink, and its structural resilience, as a result of the stronger interactions between the ionomer and catalyst, was 29.2 % higher than that of I-ink. For the LSC ink, the addition of propionic acid reduced the surface tension of the original solvent, thereby suppressing the “coffee ring” effect. This creates a thin, uniform catalyst deposition layer. Based on this, the ECSA of electrodes derived from N-ink was 62.7 m^2^∙g_Pt_^−1^, but the parameter for P-ink reached 112.2 m^2^∙g_Pt_^−1^. Therefore, the inhibition of impurity generation in HSC ink will provide better coatability, but the introduction of impurities will promote the uniform deposition of the catalyst in LSC ink. In the end, extending this study to the industrial scale will be particularly valuable for the improvement of the electric generation performance of PEMFCs, which is accessible by controlling of the HSC ink quality to establish the desired catalyst layer microstructure.

## Figures and Tables

**Table 1:** Sample composition of catalyst inks.

Ink Type	Pt/C (g)	Deionized Water (g)	1-Propanol (g)	Nafion^®^ Solution (g)	Solid Content (%)
HSC ink	5.7	14.082	13.705	20.517	12.45
LSC ink	0.002	0.000	0.967	0.0323	0.20

**Table 2:** Summary of impurity concentrations of the catalyst inks derived from GC-MS data.

Sample	Acetic Acid	Propionic Acid	Propyl Propionate
I-ink	0.0898 wt.%	0.0224 wt.%	0.00046 wt.%
D-ink	0.0025 wt.%	0.0126 wt.%	0.0003 wt.%

**Table 3:** Current densities of oxidation peaks recorded from the CV of NPA and EA.

Condition	NPA’s Oxidation Current Density/mA cm^−2^			EA’s Oxidation Current Density/mA cm^−2^		
	Peak I	Peak II	Peak III	Peak I	Peak II	Peak III
N_2_-25 °C	0.855	3.66	1.338	2.046	3.506	3.231
O_2_-25 °C	1.266	4.148	1.758	2.634	4.241	3.532

**Table 4:** Calculated Herschel–Bulkley parameters for the catalyst inks.

Ink	$\mathrm{Yield}\;\mathrm{Stress},\;\sigma_{0}\;(\mathrm{Pa})$	$\mathrm{Consistency}\;\mathrm{Index},\;\kappa (\mathrm{Pa}\cdot s^{n})$	Flow Index, n	*R* ^2^
I-ink	2.49505	0.08131	0.76772	0.99982
D-ink	1.6832	0.05865	0.80801	0.99971

**Table 5:** Properties of electrodes deposited by N-ink and P-ink.

Sample	ECSA (m^2^∙g_Pt_^−1^)	MA (mA∙mg_Pt_^−1^)
P-ink	112.2	84.4
N-ink	62.7	60.5

## Data Availability

Not applicable.

## Supplementary Materials

The following supporting information can be downloaded at: https://www.mdpi.com/article/10.3390/membranes12050541/s1, Figure S1: Total ion current (TIC) spectrum of D-ink; Figure S2: Cyclic voltammograms recorded with Pt/C electrode in 0.05 M H_2_SO_4_ + 0.1 M NPA solution (a) and 0.1 M EA solution (b) with a scan rate of 20 mV·s^−1^ under N_2_ atmosphere. Figure S3: A side view of catalyst ink droplets at the initial state and top view of ink droplets with the evolution of time for N-ink (a) and P-ink (b).
