# Supplementary material for: Influence of Degassing Treatment on the Ink Properties and Performance of Proton Exchange Membrane Fuel Cells
Source: Membranes (Basel). 2022 May 22;12(5):541. doi: 10.3390/membranes12050541 (PMC9145345; doi:10.3390/membranes12050541)
Supplement: Supplementary file 1 [file membranes-12-00541-s001.zip › membranes-1672762-supplementary.pdf]

Supporting information of

Influence of Degassing Treatment on the Ink Properties and Performance of Proton Exchange Membrane Fuel Cells

Pengcheng Liu, Daijun Yang,* Bing Li, Pingwen Ming, and Cunman Zhang

Clean Energy Automotive Engineering Center, School of Automotive Studies, Tongji University, Shanghai 201804, China

* Corresponding author: Daijun Yang

E-mail: yangdaijun@tongji.edu.cn

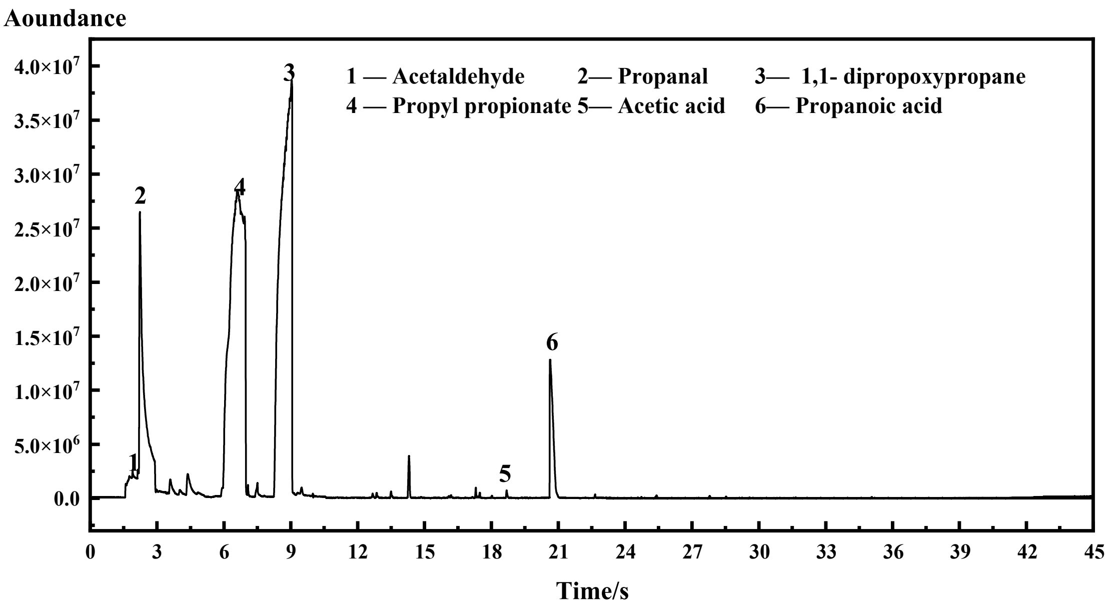

Figure S1. Total ion current (TIC) spectrum of D-ink

Electrochemical test for oxidation behavior of n-propanol and ethyl alcohol

All electro-oxidation experiments were performed based on electrochemical cells at 25 °C. Electrolyte solutions were prepared using ultrapure water and n-propanol or ethyl alcohol dissolved in 0.1 M alcohol + 0.05 M H_2SO_4. These solutions were de-aerated with O_2 or N_2. The electro-oxidation experiments system comprised of a thin film of catalyst ink deposited on a glassy carbon substrate as the working electrode (WE), a reversible hydrogen electrode (RHE) as the reference electrode, and a platinum sheet as the counter electrode. All electrochemical measurements were carried out with an electrochemical workstation (CHI. Instrument company, 760E). To prepare a working electrode, 10 μL ink was transferred to the RDE ($S = 0.196\ cm^2$) and naturally dried. The data of CV was recorded at a potential range from 0.01 V to 1.45 V with the scanning rate of $0.02\ V\cdot s^{-1}$ in the electrolyte solution.

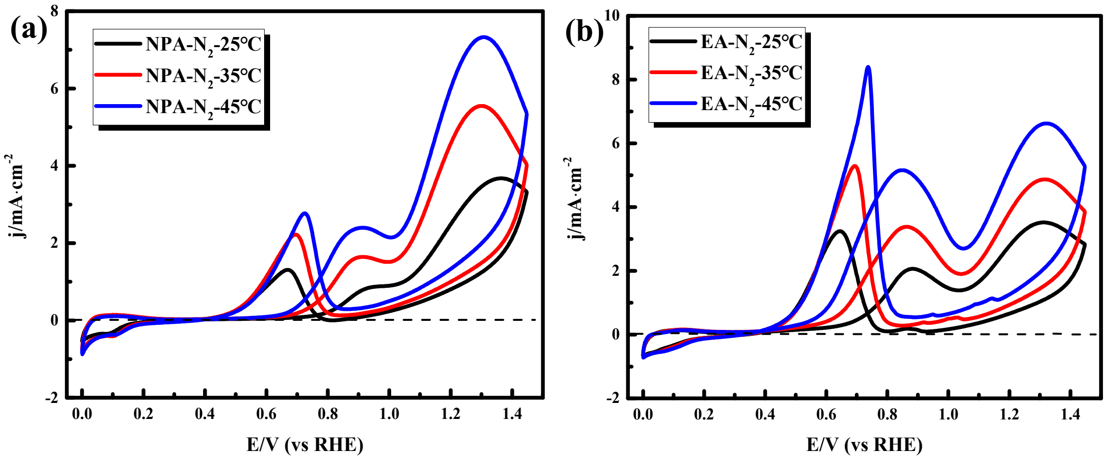

Figure S2. Cyclic voltammograms recorded with Pt/C electrode in 0.05 M H_2SO_4 + 0.1 M NPA solution (a) and 0.1 M EA solution (b) with a scan rate of $20\ mV\cdot s^{-1}$ under N_2 atmosphere.

The effects of reaction temperature on the electro-oxidation of NPA and EA were discussed. Previous researches have shown that propanol, propionic acid, and CO_2 are

the main electro-oxidation products of NPA, whereas acetic acid, aldehyde, and CO_2 are produced from EA[1-3]. Results shown in Figure S2 indicate that as the reaction temperature increased, the intensity of oxidation current increases significantly. This shows the importance of maintaining a low temperature during ink preparation and storage procedure.

[1] Pastor E, Wasmus S, Iwasita T, Arévalo MC, González S, Arvia AJ. Spectroscopic investigations of C3 primary alcohols on platinum electrodes in acid solutions. J Electroanal Chem. 1993;350:97-116.

[2] Umeda M, Sugii H, Uchida I. Alcohol electrooxidation at Pt and Pt–Ru sputtered electrodes under elevated temperature and pressurized conditions. J Power Sources. 2008;179:489-96.

[3] Kunfang Tu GL, Yanxia Jian. Effect of Temperature on the Electrocatalytic Oxidation of Ethanol. Acta Phys -Chim Sin. 2020;36:1906026.

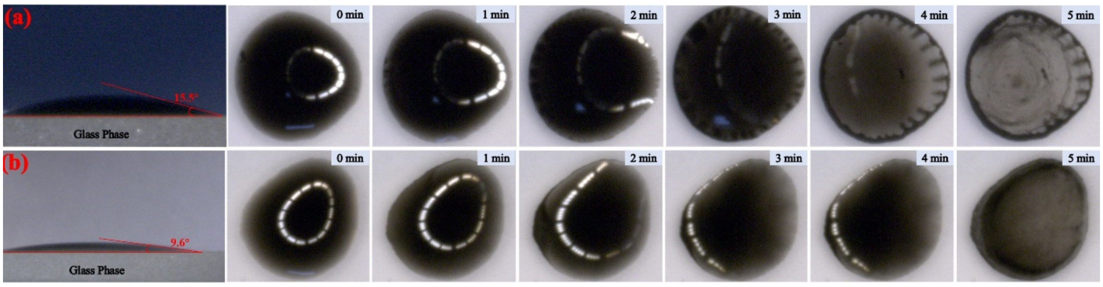

Figure S3. A side view of catalyst ink droplets at the initial state and top view of ink droplets with the evolution of time for N-ink (a) and P-ink (b)

As shown in Figure S3a, the catalyst is enriched at the edge of the droplet as the solvent continues to evaporate. The same phenomenon is illustrated in Figure S3b. Notably, the introduced propionic acid has a higher boiling point and lower surface tension than the original solvent. The droplet of P-ink has a smaller initial contact angle of 9.6° compared to that of N-ink (15.5°). The contact angle rapidly decreases as the surface tension declines. As evaporation proceeds, the water evaporation rate at the edge of the droplet exceeds that at the center, whereas evaporation rate of the propionic acid at the edge of the droplet is slower. Therefore, propionic acid gradually becomes enriched at the edge. The difference in surface tension between the edge and center of the droplet creates Marangoni effect[1]. On this basis, enhanced Marangoni flow moves the catalyst particles radially from edge to the center of droplet surface, unlike the outward capillary flow, thereby inhibiting the “coffee ring” effect. Similarly, Moon et al[2]. employed higher boiling point solvent with lower surface tension, ethylene glycol, to induce the formation of a uniform deposit of silver nanoparticles due to surface tension gradient-induced inward Marangoni flow. This implies that impurities of LSC ink had a positive effect on the formation of WE.

[1] Hu H, Larson RG. Marangoni effect reverses coffee-ring depositions. J Phys Chem B. 2006;110:7090-4.

[2] Kim D, Jeong S, Park BK, Moon J. Direct writing of silver conductive patterns: Improvement of film morphology and conductance by controlling solvent compositions. Appl Phys Lett. 2006;89.
